# Transcriptional Programs Underlying Cold Acclimation of Common Carp (*Cyprinus carpio* L.)

**DOI:** 10.3389/fgene.2020.556418

**Published:** 2020-09-23

**Authors:** Yong Long, Xixi Li, Fengyang Li, Guodong Ge, Ran Liu, Guili Song, Qing Li, Zhigang Qiao, Zongbin Cui

**Affiliations:** ^1^State Key Laboratory of Freshwater Ecology and Biotechnology, Institute of Hydrobiology, Chinese Academy of Sciences, Wuhan, China; ^2^University of Chinese Academy of Sciences, Beijing, China; ^3^Guangdong Provincial Key Laboratory of Microbial Culture Collection and Application, State Key Laboratory of Applied Microbiology Southern China, Guangdong Institute of Microbiology, Guangdong Academy of Sciences, Guangzhou, China; ^4^College of Fisheries and Life Science, Dalian Ocean University, Dalian, China; ^5^Fisheries College, Henan Normal University, Xinxiang, China

**Keywords:** cold acclimation, common carp, stress response, transcriptional program, gene expression, genetic diversity, alternative splicing

## Abstract

Properly regulated transcriptional responses to environmental perturbations are critical for the fitness of fish. Although gene expression profiles in the tissues of common carp upon cold stress were previously characterized, the transcriptional programs underlying cold acclimation are still not well known. In this study, the ability of three common carp strains including Hebao red carp (HB), Songpu mirror carp (SPM) and Yellow river carp (YR) to establish cold resistance after acclimation to a mild hypothermia stress at 18°C for 24 h was confirmed by measurements of the critical thermal minimums (CTMin). The gene expression profiles of the brain and the heart from these strains under both control and cold-acclimated conditions were characterized with RNA-sequencing. The data of the three common carp strains with different genetic background were combined in the differential gene expression analyses to balance the effects of genetic diversity on gene expression. Marked effects of tissue origins on the cold-induced transcriptional responses were revealed by comparing the differentially expressed gene (DEG) lists of the two tissues. Functional categories including spliceosome and RNA splicing were highly enriched in the DEGs of both tissues. However, steroid biosynthesis was specifically enriched in DEGs of the brain and response to unfolded protein was solely enriched in DEGs of the heart. Consistent with the up-regulation of the genes involved in cholesterol biosynthesis, total cholesterol content of the brain was significantly increased upon cold stress. Moreover, cold-induced alternative splicing (AS) events were explored and AS of the *rbmx* (RNA-binding motif protein, X chromosome) gene was confirmed by real-time quantitative PCR. Finally, a core set of cold responsive genes (CRGs) were defined by comparative transcriptomic analyses. Our data provide insights into the transcriptional programs underlying cold acclimation of common carp and offer valuable clues for further investigating the genetic determinants for cold resistance of farmed fish.

## Introduction

Common carp is one of the most extensively farmed fish around the world, which contributes up to 10% (4.129 million tons in 2017) of global annual freshwater aquaculture production (FAO, 2017). Except for the importance in aquaculture, common carp is well characterized for its genetic diversity. Common carp has an allotetraploid genome (Xu et al., 2014b) and the origin of the two subgenomes was recently elucidated (Xu et al., 2019). During the history of domestication for more than 2000 years, it has been bred into numerous strains and local populations (Xu et al., 2014b). For example, dozens of domesticated strains, populations as well as many hybrid lines are currently cultured in China, such as Songpu mirror carp, Hebao red carp, Xingguo red carp, Yellow river carp, Heilongjiang carp, Jian carp, Koi carp, and Oujiang color carp (Xu et al., 2012, 2014a,b; Wang et al., 2016). These strains demonstrate morphological diversities including body colors, scale patterns and body shapes, and differ in growth rate and tolerance to environmental stresses (Xu et al., 2014b). The genetic diversity in the genome of common carp offers valuable genetic resources for selective breeding to improve economically important traits.

Except for the visible and measurable traits, the responses and resistance to environmental stresses have emerged as important indexes for fish breeding, which represent a crucial component of both survival and fitness. Understanding the mechanisms determining stress responses offers new opportunities for breeding fish strains with an increased tolerance to environmental perturbations (Gracey et al., 2004). Hypothermia is a frequently encountered environmental stress for ectotherms like fish, which adversely influences the production of fishery. Extensive mortality of many farmed fish species could be caused by cold weathers in the winter (Song et al., 2019). The transcriptional responses to cold stress have been characterized in numerous model and farmed fish species, such as zebrafish (*Danio rerio*) (Long et al., 2012, 2013, 2015), orange-spotted groupers (*Epinephelus coioides*) (Sun et al., 2019), blue tilapia (*Oreochromis aureus*) (Nitzan et al., 2019), pufferfish (*Takifugu fasciatus*) (Wen et al., 2019), yellow drum (*Nibea albiflora*) (Xu et al., 2018), and Atlantic killifish (*Fundulus heteroclitus*) (Healy et al., 2017). These studies shed light on the mechanisms underlying cold acclimation and the resistance to lethal cold stress in fish.

Common carp is well known for its robustness against cold stress, which can survive several months of exposure to low temperatures of 0–4°C (Liang et al., 2015). A previous study characterized the transcriptional responses in different tissues of common carp to cold stress using microarrays containing 13,440 cDNA probes (Gracey et al., 2004). The transcriptional responses of Amur carp (*Cyprinus carpio* haematopterus), an indigenous population of common carp lives in the Amur River, were profiled based on a *de novo* transcriptome assembly (Liang et al., 2015). Our recent study characterized and compared the cold-induced transcriptional responses in the larvae of two closely related carp species with distinct cold resistance (Ge et al., 2020). These studies furthered our understanding of the molecular mechanisms underlying the establishment of cold resistance in common carp. However, the transcriptional programs triggered by cold stress are not well known for common carp due to the limitations in the research methods, transcriptome annotations, and developmental stages and tissue types of samples in the previous studies. Considering the marked genetic diversity in the genome of common carp and the effects of genetic background on the responses to environmental stresses, investigating and comparing the transcriptional responses in multiple strains, tissues and developmental stages would help to define the core genetic programs underlying cold acclimation.

In this study, the capacity of three common carp strains including Hebao red carp (HB), Songpu mirror carp (SPM) and Yellow river carp (YR) to establish cold resistance upon a mild cold stress (18°C) was confirmed. The brain and heart transcriptome profiles of the control and cold-acclimated fish of different strains were characterized. Brain and heart were selected because their functionalities are sensitive to changes in environmental temperatures and are paramount for fish survival from acute hypothermal stress (Tseng et al., 2014; Keen et al., 2017). The gene expression datasets were analyzed by functional enrichment analyses to extract insights into the cold-regulated biological processes and pathways. The genes underwent cold-induced alternative splicing (AS) were also identified. Finally, a core set of CRGs for carp were defined by comparing the DEG lists of this study with those of our previously published research investigating cold-induced transcriptional responses in larvae of the SPM carp and the Barbless carp (*Cyprinus pellegrini*) (Ge et al., 2020). The Barbless carp are sensitive to lethal cold stress (Sun and Liang, 2004); although they maintain the ability to increase cold resistance upon a mild hypothermia preconditioning, both their basal and enhanced cold resistance were lower than those of the SPM common carp (Ge et al., 2020). These datasets were selected for comparison because they were generated using the same version of reference genome and annotation, and the samples were exposed to the same treatment regime. Our data revealed the critical transcriptional programs underlying cold acclimation in the brain and the heart, and defined a core set of CRGs for the carp species. The data provided here offer valuable clues for investigating the key genes and the genetic variants regulating cold resistance of common carp.

## Results

### Cold Acclimation of the Common Carp Strains

**FIGURE 1:**
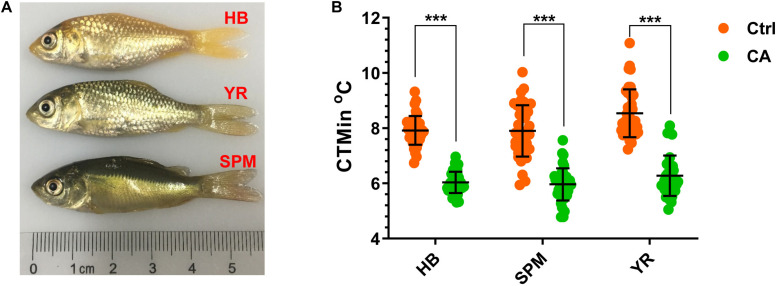
Cold acclimation of the three common carp strains. **(A)** Photos of the experimental fish. HB, Hebao red carp; YR, Yellow river carp; SPM, Songpu mirror carp. **(B)** Critical thermal minimums (CTMin) of the control (Ctrl, maintained at 28^*o*^C) and the cold-acclimated (CA, exposed to 18^*o*^C for 24 h) fish. Individual cases are displayed, the mean and SD of data sets are indicated (*n* = 50); ***p < 0.001.

Critical thermal minimum (CTMin) is a widely used index for the cold tolerance of fish (Bennett and Judd, 1992; Currie et al., 1998; Rajaguru and Ramachandran, 2001; Rajaguru, 2002; Ford and Beitinger, 2005), which is determined by decreasing the water temperature at a constant rate; and the CTMin of an animal is the temperature at which it loses the ability to maintain equilibrium (Currie et al., 1998). This method is fast, requires few fish, does not confuse handling stress with thermal stress, and approximates natural conditions better than the static methods (Bennett and Judd, 1992; Currie et al., 1998). The CTMin of both the control (Ctrl, maintained at 28°C) and cold-acclimated (CA, exposed to 18°C for 24 h) fish were measured to evaluate the effects of CA on cold tolerance. Representative photos of the experimental fish are displayed in Figure 1A. After acclimation at 18°C for 24 h, the CTMin values for the HB, SPM and YR strains were decreased from 7.92, 7.90, and 8.53°C to 6.03, 5.96, and 6.27°C, respectively (*p* < 0.001 for all the cases, Figure 1B). A general linear model analysis indicated that acclimation (CA versus Ctrl), strain (HB, SPM and YR) and body weight accounted for 61.8 % (*p* = 9.5E-25), 6.9 % (*p* = 0.02) and 5 % (*p* = 0.017) of the total deviance in CTMin, but no significant effect was observed for the interaction between strain and acclimation. Together, acclimation at 18°C for 24 h significantly increased cold resistance of all the tested common carp strains.

### Differential Gene Expression Induced by Cold Stress

The number of sequencing fragments for the samples ranged from 13.43 to 21.02 M, and a total of 401.82 M read pairs were obtained in this project (Supplementary Table 1). There was no significant difference in the mapping rate of data sets from different samples. A total of 36,874 genes were found to be expressed with a threshold of TPM (transcript per million) = 1 in both the samples of at least one tissue type (under Ctrl or CA condition) of any strain. The sample correlation matrix generated from the gene expression data indicated good correlations between the biological replicates (Pearson correlation coefficient ranged from 0.957 to 0.995, Supplementary Figure 1A). The results of the principle component analysis (PCA) demonstrated a marked discrepancy between the brain and heart samples (Supplementary Figure 1B), suggesting significant difference in gene expression.

**FIGURE 2:**
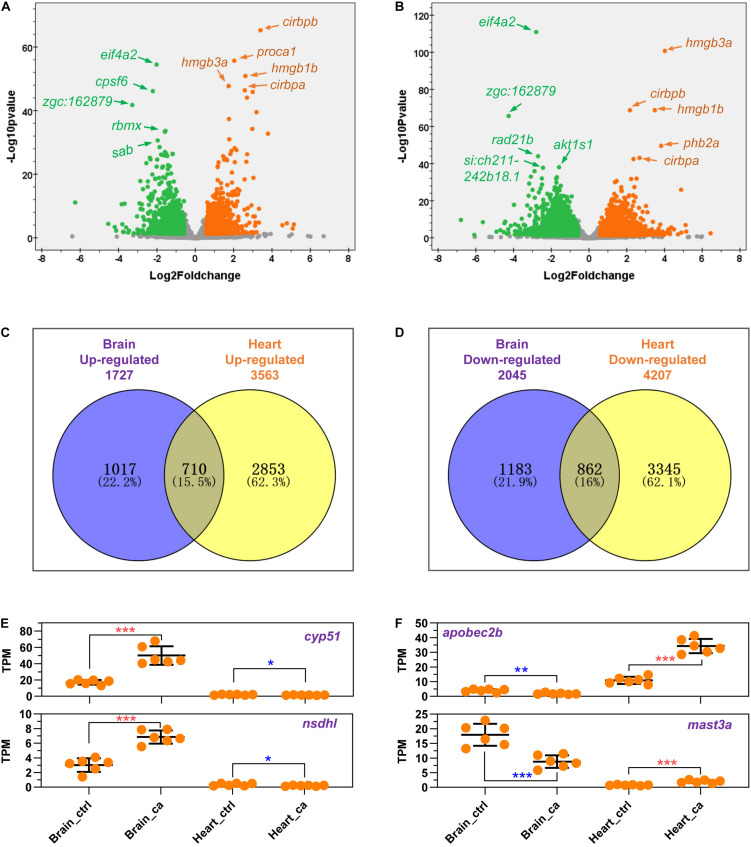
Cold-regulated gene expression in the brain and the heart of common carp. **(A,B)** Volcano plots indicate the up- and down-regulated genes in the brain **(A)** and the heart **(B)**. The top up- and down-regulated genes are shown in yellow and green, respectively. **(C,D)** Venn plots indicate numbers of the common and tissue-specific up- **(C)** and down-regulated **(D)** genes in the brain and the heart. **(E)** Genes up-regulated in the brain and down-regulated in the heart. **(F)** Genes up-regulated in the heart and down-regulated in the brain. Individual cases are displayed, the mean and SD of data sets are indicated (*n* = 6). The means of gene abundance for different treatments were compared by Independent Sample *T*-test; *p < 0.05; **p < 0.01; ***p < 0.001.

The differentially expressed genes (DEGs) in the brain and the heart are displayed in Figures 2A,B and listed in Supplementary Table 2. The most significantly up- and down-regulated genes are shown in yellow and green, respectively. The brain and the heart shared most of the top up-regulated genes such as *cirbpa*, *cirbpb*, *hmgb1*, and *hmgb3a*. These genes were suggested to be the marker genes for cold stress by previous studies (Gracey et al., 2004; Long et al., 2012, 2013). The top down-regulated genes shared by the two tissues included *eif4a2* and zgc:162879.

The total numbers of the up- and down-regulated genes for the two tissues are displayed in Figures 2C,D. Upon cold stress, the heart demonstrated more DEGs than the brain. The numbers of both the up- and down-regulated genes for the heart were approximately double of those for the brain, suggesting a larger extent of transcriptional responses to the cold stress. A total of 710 up- and 862 down-regulated genes were shared by the two tissues, representing the common responses of the brain and the heart to cold stress. Moreover, more than half of the DEGs were tissue-specific, indicating the significant effects of tissue type on the responses to cold stress.

Interestingly, a subset of genes demonstrated opposite responses to cold stress in the brain and the heart (Supplementary Table 3). The representatives are shown in Figures 2E,F. Genes such as *cyp51* and *nsdhl* were up-regulated in the brain but down-regulated in the heart (Figure 2E); while *apobec2b* and *mast3a* were up-regulated in the heart but down-regulated in the brain (Figure 2F). These genes represent the opposite metabolic regulations elicited by cold stress in the different tissues.

### Validation of the RNA-Seq Data by Quantitative Real-Time PCR

The expression of genes such as *eef2b* (eukaryotic translation elongation factor 2b), *jarid2b* (jumonji, AT rich interactive domain 2b), *rbm5* (RNA binding motif protein 5) and *ciarta* (circadian associated repressor of transcription a) was analyzed by quantitative real-time PCR (qPCR) to validate the results of RNA-seq. These genes were up- or down-regulated in all the samples. As shown in Table 1, up- or down-regulation of all the genes were confirmed by qPCR. A Pearson correlation analysis revealed the significant correlation (*p* = 1.46E-18) between the log2Foldchange of gene expression obtained by RNA-seq and qPCR (Supplementary Figure 2). The results of qPCR assays indicate the validity of the RNA-seq data.

**TABLE 1:** Validation of RNA-seq results using qPCR.

	HB-brain		SPM-brain		YR-brain		HB-heart		SPM-heart		YR-heart	
Gene name	RNA-seq	qPCR	RNA-seq	qPCR	RNA-seq	qPCR	RNA-seq	qPCR	RNA-seq	qPCR	RNA-seq	qPCR
*eef2b*	1.08	0.79**	1.12	0.84**	1.34	1.08**	1.48	0.85**	2.08	0.85**	2.57	0.91**
*jarid2b*	1.73	0.89**	0.71	1.01**	1.47	1.31**	1.99	0.79**	1.51	0.80**	2.40	0.86**
*lancl1*	1.12	0.68*	1.50	0.83**	1.02	0.75**	2.47	1.10**	4.15	1.12**	2.15	0.73**
*mrps34*	1.21	0.80**	1.76	0.66*	1.55	1.27**	1.99	1.95**	2.16	1.41**	1.92	1.22**
*rbm5*	–1.87	−1.09**	–1.68	−1.11**	–1.96	−0.74**	–1.65	−1.50**	–1.65	−1.12**	–1.42	−1.19**
*clk4a*	–1.96	−1.90**	–2.04	−1.52**	–2.06	−1.69**	–1.55	−1.19**	–0.89	−1.26**	–1.41	−0.88**
*cbx1a*	–1.99	−0.66*	–3.46	−0.74*	–2.24	−0.67*	–2.61	−0.79**	–2.85	−0.83**	–1.55	−0.62*
*cyp2ad6*	–2.61	−2.27**	–1.72	−1.23**	–2.64	−2.07**	–2.77	−1.99**	–2.67	−1.73**	–1.37	−1.86**

The data indicate mean log2Foldchange of gene expression between the Ctrl and CA samples. Significance of difference (between Ctrl and CA) in gene expression analyzed by qPCR is displayed. *p < 0.05; **p < 0.01.

### GO and KEGG Enrichments for the DEGs

**FIGURE 3:**
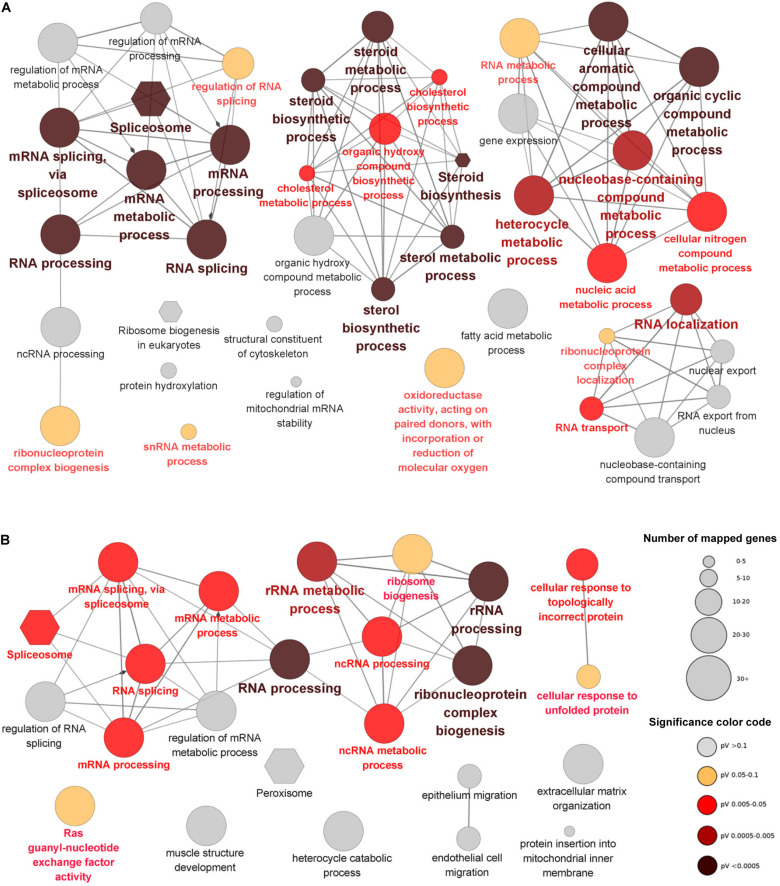
GO and KEGG enrichments for the DEGs identified from the brain **(A)** and the heart **(B)**. The nodes represent the enriched GO (ellipse) and KEGG (hexagon) terms and the edges between nodes indicate the existence of shared genes. The size and color of nodes represent the number of associated genes and significance level of the functional terms.

GO and KEGG enrichment analyses were performed to provide insights into the functions of the DEGs. The DEGs of the brain were mainly enriched in 4 functional groups including RNA processing, steroid metabolic process, cellular aromatic compound metabolic process and RNA localization (Figure 3A). Most of the DEGs mapped to spliceosome (KEGG pathway) were up-regulated and all the members of the EJC/TREX complex were induced by cold stress (Supplementary Figure 3). Furthermore, all the genes encoding the enzymes catalyzing the synthetic reactions from terpenoid backbone to cholesterol were up-regulated (Supplementary Figure 4). Separate functional terms such as oxidoreductase activity, snRNA metabolic process and fatty acid metabolic process were also enriched in the brain DEGs.

Like the situations of brain, the DEGs of heart were also enriched in the functional groups associated with RNA processing and mRNA splicing via spliceosome. Functional terms such as cellular response to topologically incorrect protein, Ras guanyl-nucleotide exchange factor activity, peroxisome and extracellular matrix organization were also enriched in the DEGs of heart. However, no functional term associated with steroid metabolic process was found for the DEGs of heart, suggesting the specificity of this process in brain upon cold stress (Figure 3B).

### Functional Gene Sets Enriched in the Whole Gene Lists

The gene set enrichment analysis (GSEA) offers the flexibility to extract further biological insights from the whole gene expression datasets. This method analyzes the rank of all the genes in the list and is not limited by the arbitrary threshold for the identification of DEGs (fold change and *p*-value) (Subramanian et al., 2005).

**FIGURE 4:**
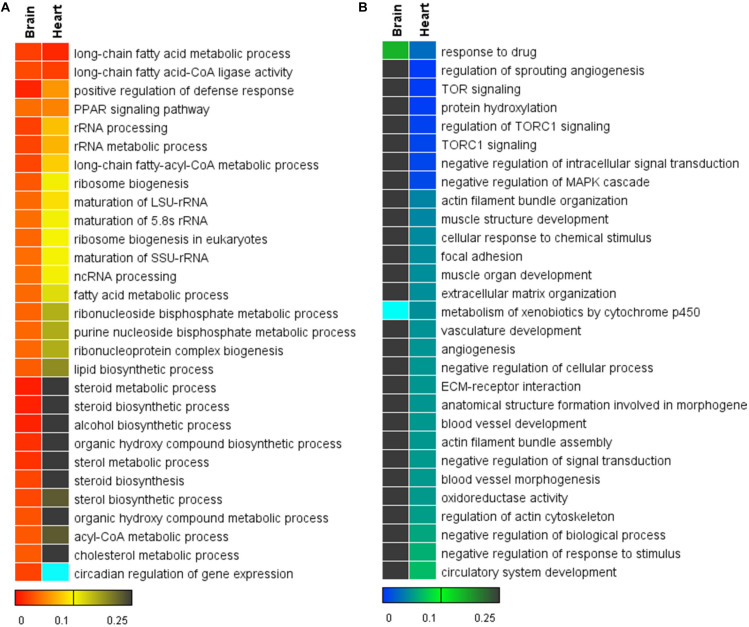
Heat maps for the top 30 functional gene sets enriched in the whole gene expression datasets. **(A)** Up-regulated gene sets. **(B)** Down-regulated gene sets. The color scales indicate the significance level of gene sets. The empty values are shown in cyan.

The top gene sets (GO biological processes and KEGG pathways) enriched for the brain and the heart are displayed in Figures 4A,B. Gene sets associated with long-chain fatty acid biosynthesis, PPAR signaling pathway, ncRNA processing and ribosome biogenesis were up-regulated in both tissues. Nearly all the identified gene sets demonstrated higher statistical significance in the brain than the heart. Consistent with the results of the enrichment analyses for the DEGs, the GSEA also identified the up-regulation of gene sets associated with steroid biosynthesis in the brain but not in the heart (Figure 4A). Furthermore, the GSEA identified the up-regulation of circadian regulation of gene expression for the brain. It was not found to be enriched in the DEGs.

There were many significantly down-regulated gene sets for the heart upon cold stress, such as regulation of sprouting angiogenesis, TOR signaling and protein hydroxylation (Figure 4B). Except for the gene set of response to drug, all the significantly down-regulated gene sets of the heart were not significant for the brain. Down-regulation of the gene sets associated with blood vessel development, angiogenesis and muscle development suggests that the normal functions of the heart was compromised upon cold stress.

Together, the brain had more up-regulated gene sets than the heart and the heart had more down-regulated gene sets than the brain; the GSEA identified new functional gene sets, which can complement the enrichment analyses for the DEGs.

### Leading-Edge Genes for the Representative Gene Sets

**FIGURE 5:**
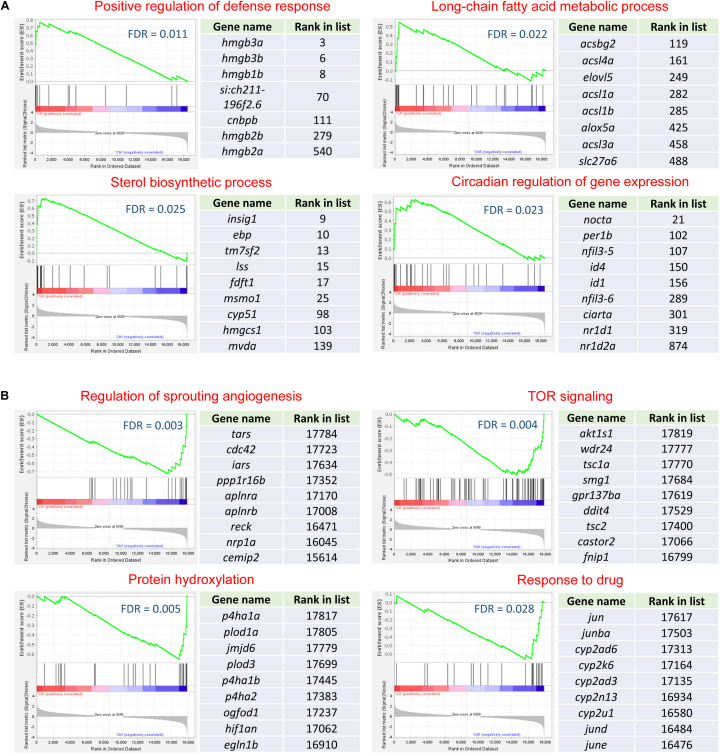
Representative gene sets and their leading-edge genes. **(A)** Up-regulated gene sets for the brain. **(B)** Down-regulated gene sets for the heart. For each gene set, the enrichment plot, the leading-edge genes and their rank in the total gene list are shown.

The representative up-regulated gene sets of the brain are displayed in Figure 5A. The leading-edge genes associated with positive regulation of defense response are mainly members of the high mobility group box family, including *hmgb3a*, *hmgb3b*, *hmgb1b*, *hmgb2a*, and *hmgb2b*. Some of these high mobility group box genes were highly ranked (top 10) in the whole gene list, suggesting the significance of their up-regulation in cold acclimation. The top genes involved in long-chain fatty acid metabolic process are mainly members of the Acyl-CoA synthetase long chain family, including *acsl1a*, *acsl1b*, *acsl3a*, and *acsl4a*; and *acsbg2*, which is a member of the acyl-CoA synthetase bubblegum family. The genes involved in sterol biosynthetic process encode the enzymes in the cholesterol biosynthesis pathway. Circadian regulation of gene expression has been reported to be affected by temperature, and the involved genes such as *per1b*, *nfil3-5*, *nr1d1*, and *nr1d2a* were previously found to be induced by cold stress in zebrafish (Long et al., 2012, 2013).

Figure 5B illustrates the representative down-regulated gene sets of the heart. In the organisms, the new blood vessels are formed through angiogenesis. The leading-edge genes involved in regulation of sprouting angiogenesis include *tars*, *cdc42*, *iars*, and *ppp1r16b*, etc. TOR (target of rapamycin) signaling is involved in the control of cell growth and proliferation. Genes such as *akt1s1*, *wdr24*, and *tsc1a* are the leading-edge genes of this gene set. Protein hydroxylation is a post-translational modification which can affect protein stability and protein-protein interaction (Zurlo et al., 2016). The down-regulation of the genes with dioxygenase activity, like *p4ha1a*, *plod1a*, *jmjd6*, and *p4ha1b* may help to prevent protein degradation in the heart upon cold stress. The leading-edge genes involved in response to drug comprise the *jun* family transcription factors and the cytochrome P450 family members.

### Cold-Induced Cholesterol Accumulation in the Brain of Common Carp

**FIGURE 6:**
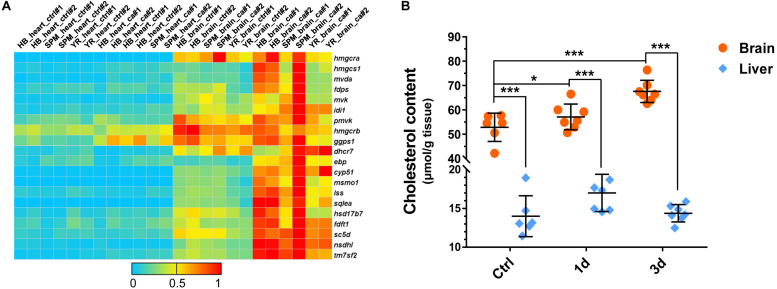
Enhanced total cholesterol accumulation in the brain of common carp during cold acclimation. **(A)** Up-regulation of genes involved in the cholesterol biosynthesis pathway upon cold stress. The color scale indicates relative gene expression level (ratio to the highest level for each gene). **(B)** Cold acclimation increased the concentration of total cholesterol in the brain of SPM carp. Data are means ± SD (*n* = 7); *p < 0.05; ***p < 0.001.

The cholesterol biosynthetic process was highly enriched within the up-regulated genes in the brain (Figures 3A, 4A). The abundances of the genes involved in cholesterol biosynthesis were higher in the brains than in the hearts, and almost all the genes were up-regulated in the brains of all the strains under cold stress (Figure 6A). To investigate whether the up-regulated gene expression would lead to an increased tissue accumulation of cholesterol, the total cholesterol concentrations in the brain and the liver of the SPM strain before and after CA were analyzed. The cholesterol content of the brain was significantly higher than that of the liver. The brain cholesterol content was significantly increased after exposure to cold stress; however, the cholesterol content of the liver was not affected by cold stress (Figure 6B). These results suggest the association of the enhanced cholesterol biosynthesis in the brain of carp with the acclimation to hypothermia stressor.

### Cold-Induced Differential Splicing of the Carp Genes

**FIGURE 7:**
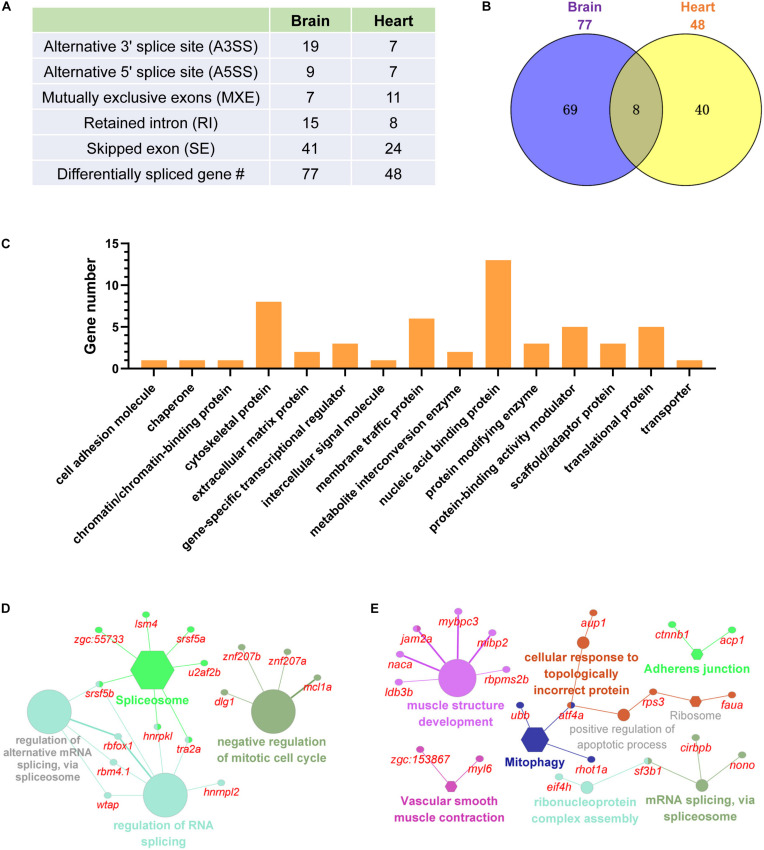
Cold-induced differential splicing of genes in the brain and the heart of common carp. **(A)** Numbers of different type of alternative splicing events. **(B)** Common and tissue-specific differentially spliced genes. **(C)** Classification of the differentially spliced genes according to the PANTHER protein class annotation. **(D,E)** Functional classification of the differentially spliced genes of the brain **(E)** and heart **(E)**. The differentially spliced genes and their associated functional terms are shown.

Except for differential expression, cold-induced differential splicing of genes in the brain and the heart were identified and compared to delineate the post transcriptional regulations associated with cold acclimation. The numbers of different type of alternative splicing (AS) events are displayed in Figure 7A. Skipped exon was the most abundant AS type. The total number of genes underwent cold-induced AS was 77 for the brain and 48 for the heart. Only 8 differentially spliced genes were shared between the two tissues (Figure 7B). In total, 117 genes were found to be differentially spliced upon cold exposure. The nucleic acid binding proteins is the most abundant protein class of these genes, followed by cytoskeletal proteins, protein-binding activity modulator and translational proteins (Figure 7C). Functional classification analyses revealed that the genes undergoing cold-induced AS in the brain are mainly involved in spliceosome, regulation of RNA splicing and negative regulation of mitotic cell cycle (Figure 7D). While the differentially spliced genes of the heart are mainly associated with muscle structure development, RNA splicing and vascular smooth muscle contraction (Figure 7E). Together, a dozen of genes involved in RNA splicing were differentially spliced upon cold stress. Differential splicing of genes related to muscle development and contraction in the heart represents a strategy for tissue modification aims to restore the functions of the heart upon a cold environment.

### Differential Splicing of the *rbmx* Gene Under Cold Stress

**FIGURE 8:**
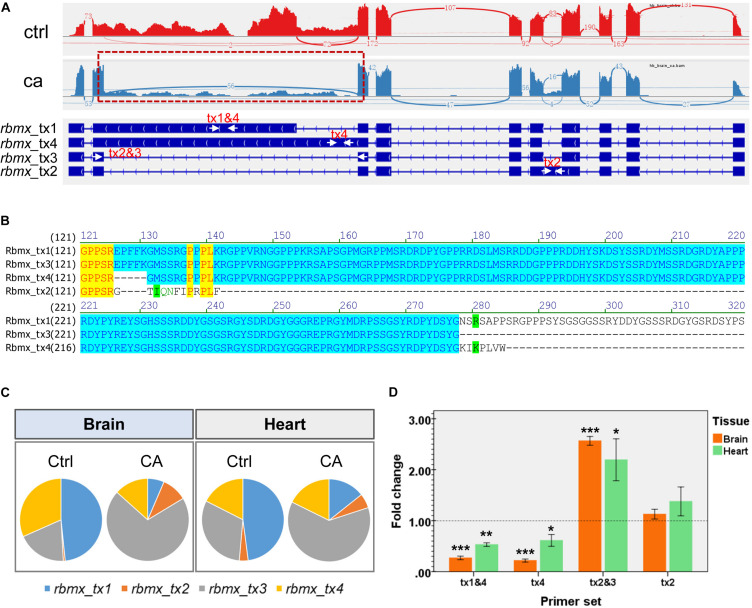
Cold-induced alternative splicing of the common carp *rmbx* gene. **(A)** Transcripts of the HB carp *rmbx* gene are displayed and the Sashimi plot indicates the number of reads split across the junctions. The arrows indicate primers used for qPCR assays to detect the abundance of transcripts. **(B)** Alignment of the peptide sequences encoded by the transcripts of carp *rbmx* gene. The identical N-terminals (1–125 amino acids) are shown in yellow. These peptides have identical N terminals and different C terminals. **(C)** Ratio of transcript abundances under control and cold acclimation conditions. **(D)** qPCR validation for the cold-induced alternative splicing of carp *rbmx* gene in tissues of the HB strain. Data are means ± SD (*n* = 3); *p < 0.05; **p < 0.01; ***p < 0.001.

The cold-induced differential splicing of the *rbmx* gene was characterized to address the significance of cold-induced alternative splicing. Expression of the *rbmx* gene was down-regulated in both the brain and the heart (Supplementary Table 2). The results of genome-guided transcript assembly indicated that the common carp *rbmx* gene had 4 transcripts. The Sashimi plots (Figure 8A) demonstrate the number of split reads and the structure of the transcripts of the *rbmx* gene. Alternative splicing of the *rbmx* gene affected the C-terminal sequences of the protein isoforms (Figure 8B). While the N-terminal sequences from all the isoforms (from 1 to 125 aa) are identical, Rbmx_tx2 has only 137 aa in length, less than half of the longest one (Rbmx_tx1, 380 aa). The results of RNA-seq indicated that *rbmx_tx1* was the most abundant transcript in the control samples; however, cold stressor significantly induced the ratio of *rbmx_tx3* (encoding 277 aa) in both the brain and heart tissues (Figure 8C). The down-regulation of *rbmx_tx1&4* and *rbmx_tx4*, and the up-regulation of *rbmx_tx2&3* were confirmed by qPCR in the brain and heart tissues of the HB strain (Figure 8D). These results indicate that the shorter isoforms of carp Rbmx are preferred upon cold stress, but the underlying mechanisms remain to be characterized.

### The Core Cold Responsive Genes (CRGs)

**FIGURE 9:**
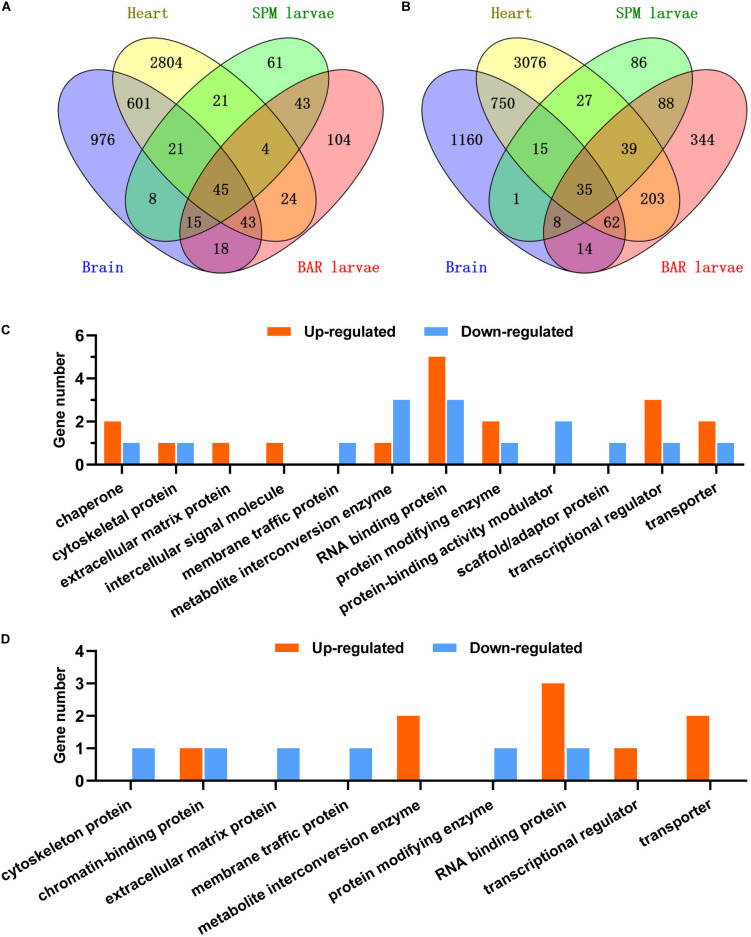
The core cold responsive genes of common carp. **(A,B)** Venn charts indicate the core set of genes up-regulated **(A)** or down-regulated **(B)** in all the experiments. Data of the brains and hearts were generated in this work, data of the SPM and BAR (Barbless carp) larvae were from our previous study (Ge et al., 2020). **(C,D)** Classification of the up-regulated **(C)** and down-regulated **(D)** core (differentially expressed in all the 4 cases) and common (differentially expressed in the cases except for BAR) genes according to the PANTHER protein class annotations (Mi et al., 2019).

The DEG lists of the brain and the heart were compared with those of the SPM and Barbless carp larvae to identify the core CRGs shared across the samples. A total of 45 core up-regulated and 35 core down-regulated genes were identified (Figures 9A,B). For the representatives of these core CRGs, genes including *cirbpa*, *cirbpb*, *hmgb1b*, *hmgb3a*, and *scd* were up-regulated; while genes such as *rbmx*, *clk4a*, *tcp11l2*, *rbm5*, and *hnrnph3* were down-regulated (Supplementary Table 4). Some of the core CRGs were previously found to be up-regulated or down-regulated by cold stresses in multiple species (Gracey et al., 2004; Long et al., 2012, 2013). The most abundant protein class annotation of the core up-regulated CRGs was RNA binding protein, followed by transcriptional regulator, transporter, protein modifying enzyme and chaperone; while the core down-regulated genes were mainly RNA binding proteins, metabolite interconversion enzymes and protein-binding activity modulators (Figure 9C). The conserved responses of these core CRGs to cold stress suggest their intimate associations with cold resistance of fish.

Furthermore, subsets of genes were found to be up-regulated (21 genes) or down-regulated (15 genes) in all the samples except for the Barbless carp larvae (Figures 9A,B). Representatives for the up-regulated genes included *ddx23*, *rbm8a*, *FO905040.1*, *chd2* and *xrn2*; while *cbx1a*, *scmh1*, *si:ch211-199b20.3*, *hsp90b1*, and *nxf1* were the representative down-regulated genes (Supplementary Table 5). The up-regulated genes were mainly classified into protein classes including RNA binding protein, transporter and metabolite interconversion enzyme; however, the down-regulated gens were not concentrated on certain functional class (Figure 9D). Taking into account the fact that the Barbless carp are sensitive to cold stress, the inability to properly regulate these common CRGs upon hypothermia stress may be related to their cold sensitivity.

## Discussion

The common carp is characterized with the economic importance and genetic diversity in the genome. The genetic diversity of common carp strains leads to distinct phenotypes in body color, body shape, scale pattern, growth rate, and response and tolerance to pathogens (Nielsen et al., 2010; Odegard et al., 2010; Jiang et al., 2014; Xu et al., 2014b; Adamek et al., 2019), offers an attractive model to dissect the genetic basis of the economic traits. The genetic background of fish also plays pivotal roles in shaping the transcriptional responses to environmental stresses, which in turn determines the resistance of the animals to the subsequent lethal level challenges of the same stress (De Nadal et al., 2011). Furthermore, common carp is an eurythermal species which can acclimate and endure large degrees of seasonal and acute temperature alterations (Sun and Liang, 2004). Most of the common carp strains can survive long time exposure to low temperature stresses (0–4°C) in the winter (Bauer and Schlott, 2004; Liang et al., 2015); however, the molecular mechanisms underlying this robustness to low temperature challenge are not well understood. Although the transcriptional responses of different tissues of cold-treated common carp were previously investigated (Gracey et al., 2004; Liang et al., 2015; Ge et al., 2020), the cold stress responses of common carp are still not fully explored due to the limitations in the gene profiling method, incompleteness of genome annotation and the specific developmental stage of the samples.

In this study, the ability of the three common carp strains including HB, SPM and YR to enhance the cold resistance upon a mild cold stressor were confirmed. The transcriptional responses of the brain and the heart of both the control and cold acclimated fish were characterized through RNA-sequencing. The three common carp strains with different genetic background were included in the analysis to balance the effects of genetic diversity on both the basal and activated gene expressions. The differentially expressed genes in the brain and the heart were identified. The comparison of the DEG lists revealed that ratios of the common DEGs between the brain and the heart were less than 20% of the total DEGs, suggesting the marked tissue specificity of transcriptional responses to cold stress. Interestingly, there were even subsets of genes demonstrated opposite responses to cold stressor in the brain and the heart. For the genes up-regulated in the brain but down-regulated in the heart, *cyp51* and *nsdhl* are involved in cholesterol metabolic process; *nocta* has 3′–5′-exoribonuclease activity and is involved in circadian rhythm. For the genes up-regulated in the heart but down-regulated in the brain, *apobec2b* has cytidine deaminase activity and is involved in cytidine to uridine editing and DNA demethylation; *mast3a* is predicted to have ATP binding, magnesium ion binding and protein kinase activity and is involved in cytoskeleton organization.

Functional enrichment analyses for the DEGs and the whole gene expression datasets of the brain and the heart revealed the common and tissue-specific processes and pathways regulated by cold stressor. RNA splicing and ribonucleoprotein complex biogenesis were enriched for the DEGs of both tissues (Figures 4A,B). These processes were previously found to be enriched in the cold-induced genes in zebrafish as well (Long et al., 2012, 2013). The processes associated with steroid biosynthesis and RNA location were specifically enriched for DEGs of the brain (Figure 4A). Steroids play pivotal roles in functions of the brain and the brain is the site of de novo steroid synthesis from cholesterol or their precursors (Do Rego et al., 2009). Steroid biosynthesis was also enriched in the DEGs in the tissues of Olive Flounder (*Paralichthys olivaceus*) exposed to low temperature stress (Hu et al., 2014). The up-regulation of genes involved in steroid biosynthesis suggests that acclimation to cold stressor requires enhanced levels of steroids for the brain cells. RNA localization is a prevalent and functionally important layer of gene regulation, which helps to increase the coherence and efficiency in the coordination of gene regulatory events (Martin and Ephrussi, 2009). Regulated localization of mRNA can facilitate rapid on-site protein production as a response to extrinsic stimuli; local translation is important for functions of neurons and axons in the brain (Chin and Lecuyer, 2017).

Cellular responses to topologically incorrect protein and unfolded proteins were significantly enriched in the DEGs of the heart (Figure 4B). Among the DEGs involved in these processes, 19 were down-regulated and 17 were up-regulated, indicating that the cold stress elicited modulation but not activation or inhibition of these processes. For example, the activating transcription factor genes *atf4a* was inhibited, while *atf6* was induced (Supplementary Table 2). ATF4 and ATF6 are the main regulators for two of the three unfolded protein response (UPR) signaling branches. Upon UPR, the translation of ATF4 is selectively increased and the enhanced accumulation of ATF4 may contribute signals to cell death pathways (Walter and Ron, 2011). ATF6 functions to increase ER protein folding capacity through regulating genes involved in protein folding, protein degradation and ER biogenesis (Walter and Ron, 2011). Therefore, the overall effect of regulation of the responses to unfolded protein in the heart during cold acclimation is to increase the ER protein folding capacity and limit the potential of apoptosis.

The functional gene set enrichment analyses (GSEA) for the whole gene sets were performed to provide further insights into the biological processes and pathways regulated by cold stress. Except for those enriched in the DEGs, new enriched functional gene sets were identified. Processes such as long-chain fatty acid metabolic process, positive regulation of defense response, PPAR signaling and ncRNA processing were significantly up-regulated in both the brain and the heart. As shown in Figure 4A, all the common up-regulated functional gene sets were more significantly enriched for the brain than the heart. For example, all the leading-edge genes of the long-chain fatty acid metabolic process (listed in Figure 5A) were significantly up-regulated in the brain, only *acsl1b*, *acsl4a*, and *elovl5* were up-regulated in the heart (Supplementary Table 2). However, the heart demonstrated larger numbers of down-regulated functional gene sets than the brain (Figure 4B). These results suggest that the brain tends to activate more biological processes, while the heart tends to limit the activities of a broad spectrum of biological functions in front of such a mild cold exposure.

Taking into account the highly significant enrichment of the steroid and cholesterol biosynthetic processes among the cold-induced genes in brain, the total cholesterol content of the brain was measured to illustrate the biological significance of the transcriptomic data. The total cholesterol content assays indicated the enhanced accumulation of total cholesterol in the brain of common carp upon cold stress. Brain is the most cholesterol-rich organ, it contains about 20% of the whole body’s cholesterol (Bjorkhem and Meaney, 2004). Cholesterol is essential for neuronal physiology, which is not only an essential structural component for cellular membrane and myelin, but also a required component for synapse and dendrite formation (Zhang and Liu, 2015). It was previously reported that increasing dietary cholesterol enhanced cold tolerance in *Drosophila melanogaster* (Shreve et al., 2007). However, the functional significance of cold-induced total cholesterol accumulation in the brain of fish remains to be further investigated.

Cold-induced differential splicing of genes was well recognized in eukaryotes (Long et al., 2013; Calixto et al., 2018). Our transcriptomic analysis identified a number of AS events for common carp upon cold stress. Again, these AS events can be classified as common and tissue-specific. Functional classification analyses revealed an array of splicing factors underwent cold-induced AS, such as *cirbpb*, *rbm4.1*, *sf3b1*, *srsf5a*, and *srsf5b*. Splicing factors play important roles in constitutive pre-mRNA splicing and alternative splicing, and also participate in post-splicing activities, such as mRNA nuclear export, nonsense-mediated mRNA decay and mRNA translation (Long and Caceres, 2009). Among these splicing factors, cold-induced AS for *Cirbp* was well characterized in mammalian tissues and cell line (Gotic et al., 2016; Horii et al., 2019) and the temperature-dependent accumulation of *Cirbp* mRNA is controlled primarily by the regulation of splicing efficiency (Gotic et al., 2016). However, functions of these splicing factors that underwent cold-induced AS during cold acclimation of fish remain unclear and deserve further investigation.

The comparative transcriptomic analysis identified the core CRGs shared across the experiments. These genes represent the conserved genetic modules underlying cold acclimation of fish. Many of them were previously found to be induced by cold stress in species like common carp and zebrafish. For example, the genes encoding the cold inducible RNA binding proteins (*cirbpa* and *cirbpb*) and the high mobility group box proteins (*hmgb1b* and *hmgb3a*) were regarded as marker genes for cold stress (Gracey et al., 2004; Long et al., 2013). CIRBP is a stress-responsive protein has protective effects against tissue injury and cell apoptosis caused by both hyperthermia and hypothermia stressors (Yu et al., 2018; Cheng et al., 2019; Liu et al., 2019). HMGB1 is the best characterized damage-associated molecular pattern, which is normally inside the cell and is released after cell death or secreted upon severe stresses. Extracellular HMGB1 triggers inflammation and adaptive immunological responses to support tissue repair and healing (Bianchi et al., 2017). These core cold-induced genes may constitute essential and conserved cellular functions to establish the cold resistance of common carp. Focusing on the functional and regulatory differences of these core CRGs between common carp and the cold-sensitive species like tilapias would provide insights into the genetic mechanisms underlying the susceptibility to hypothermal stressor.

Furthermore, we also identified common CRGs shared by samples from the cold-resistant strains but not by that of the cold-sensitive Barbless carp (Figure 9). Considering the compromised ability of the Barbless carp to establish a full strength cold-resistant phenotype upon cold acclimation, these genes may also play pivotal roles in cold tolerance. The representative up-regulated gene of this subset such as *ddx23*, *prpf31*, and *rbm8a* encode RNA binding proteins and are predicted to be involved in mRNA splicing. The potential functions of these genes are consistent with the intimate associations of regulation of RNA splicing with cold acclimation. For the down-regulate genes, *bnip3* is involved in positive regulation of programmed cell death (Ma et al., 2017). Inability of the Barbless carp to regulate these genes properly may contribute to their cold sensitivity.

## Conclusion

Exposure to a mild hypothermia stressor at 18°C for 24 h enhanced cold resistance of the three common carp strains. The transcriptome profiles of the brain and the heart of individuals from these strains under both control and cold-acclimated conditions were characterized. Functional enrichment analyses revealed the biological processes and pathways overrepresented by the DEGs. While RNA splicing and spliceosome were enriched in the DEGs of both tissues, processes associated with steroid biosynthesis were specific for the brain, and functional terms such as cellular responses to unfolded protein were specific for the heart. Gene set enrichment analyses performed with the whole gene expression datasets provided further insights into the biological functions regulated by the mild cold stressor. Long-chain fatty acid metabolic process, positive regulation of defense response and PPAR signaling were significantly up-regulated, and response to drug was down-regulated in both tissues. Except for the shared functional terms, the brain had more up-regulated and the heart had more down-regulated biological processes. Biological significance of the enrichment of cholesterol biosynthetic process in the up-regulated genes was illustrated by the increased total cholesterol content in the brain. Cold-induced AS of genes was also analyzed. In total, 117 genes were found to be differentially spliced upon cold stress. These differentially spliced genes are mainly involved in spliceosome, negative regulation of mitotic cell cycle and muscle structure development. Finally, the core set of CRGs were defined through comparative transcriptomic analyses. Genes such as *cirbpa*, *cirbpb*, *hmgb1b*, *hmgb3a*, and *scd* were representatives of the core up-regulated genes, while *rbmx*, *clk4a*, *tcp11l2*, *rbm5*, and *hnrnph3* represent the core down-regulated genes. Our data shed new light on the transcriptional responses of common carp to cold stressor and provide interesting clues to investigate the genetic determinants for the cold tolerance of farmed fish.

## Materials and Methods

### Experimental Fish

The animal protocol for this study was approved by the Institutional Animal Care and Use Committee of Institute of Hydrobiology (Approval ID: Y32A041501). Two-year-old brood fish of Hebao red carp (HB), Yellow river carp (YR), and Songpu mirror carp (SPM) were transported to and maintained in the ponds of a fish farm located in the Henan province, China. The brood fish of HB strain were obtained from Wuyuan Hebao red carp seed farm, Wuyuan, China; the SPM brood fish were obtained from Heilongjiang River Fisheries Research Institute, Chinese Academy of Fishery Sciences, Haerbin, China; the YR strain brood fish were obtained from a local fish farm at Qixian, China.

After cultivation to gonad maturation, the brood fish of different strains were injected with hormones at the same day to induce ovulation. The hormones used for induced reproduction included 500 U human chorionic gonadotropin (HCG), 2.5 mg luteinizing hormone releasing hormone (LHRH) and domperidone (DOM) at 2 mg/kg body weight for the females and half of the dosage was used for the males. Artificial fertilization was conducted by mixing the semen and oocytes of the same strain. The fertilized eggs were hatched in our laboratory with aerated tap water.

The larvae and juveniles were fed with brine shrimp nauplii and commercial floating fish feed, respectively. The fish were raised in tanks supplied with circulating water. The water temperature of the aquarium system was 28 ± 0.5°C. Three-month-old juveniles were used for subsequent experiments. Average body weights for the experimental fish of HB, SPM and YR strains were 2.79 ± 0.71 g, 2.88 ± 0.63 g and 2.88 ± 0.85 g, respectively. No significant difference in the body weight was detected between different strains.

### Cold Acclimation and CTMin Determination

For cold acclimation, the fish of different strains were directly transferred from 28 to 18°C and maintained for 24 h. Water temperature was controlled by using three Thermo Scientific^TM^ ARCTIC PC200 A40 Refrigerated Circulators. The fish were plunged into a homemade cage fitting the chamber of the circulators. For CTMin measurement, the water temperature was decreased 0.3°C/min from the acclimated temperature (28 or 18°C). When the CTMin was reached (loss of equilibrium), the fish was taken out and weighted.

### Total RNA Extraction

The control and cold-acclimated fish were euthanized with 300 mg/L tricaine methanesulfonate (MS 222) before dissection. The brain and heart were collected for total RNA extraction. The brains were treated separately and hearts from three fish of the same strain and treatment were combined because the tissue amount was small. The tissue samples were homogenized in TRIzol (Invitrogen) using a TissueRuptor II from QIAGEN. Integrity of the RNA samples was checked by agarose electrophoresis. Concentration of the RNA samples was measured by using a Quawell 5000 UV-Vis Spectrophotometer.

### Library Construction and High-Throughput Sequencing

The experimental design contained duplicates of 12 samples from 3 carp strains (HB, SPM and YR), 2 conditions (Ctrl and CA) and 2 tissues (brain and heart). In total, 24 sequencing libraries were constructed and sequenced. Library construction and high-throughput sequencing were performed by experts of the Analytical & Testing Center at Institute of Hydrobiology, Chinese Academy of Sciences^1^. Briefly, 1 mg of DNase I-treated total RNA of each sample was used for sequencing library construction. The NEBNext^®^ rRNA Depletion Kit was used for rRNA depletion. The RNAs were purified by using the Agencourt RNAClean XP Beads from Beckman Coulter. RNA fragmentation, first strand and second strand cDNA synthesis and double-stranded cDNA end repair were performed using the NEBNext^®^ Ultra^TM^ Directional RNA Library Prep Kit for Illumina^§^. Double strand cDNAs were purified using the Agencourt AMPure XP from Beckman Coulter and ligated to adaptors of the NEBNext Multiplex Oligos for Illumina. Finally, the Q5 Hot Start HiFi PCR Master Mix (NEB) was used for PCR enrichment of the adaptor-ligated DNA. Concentration and quality of the libraries were measured by using the Agilent High Sensitivity DNA Kit and a Bioanalyzer 2100 from Agilent Technologies.

The libraries were sequenced using a NextSeq 500 system according to the standard Illumina protocols. All the libraries were sequenced for 150 nt at both ends. The sequencing data produced in this study have been deposited in the NCBI Sequence Read Archive (SRA) under the BioProject accession number PRJNA562102.

### Reads Preprocessing

The quality of the raw sequencing reads was analyzed using fastqc^2^ and multiqc (Ewels et al., 2016). The raw reads were trimmed and filtered using PRINSEQ (version 0.20.4) (Schmieder and Edwards, 2011). At the first step, low score (Phred quality score < 20) and ambiguous bases (N) were trimmed from both ends of the reads. Then, the trimmed reads were filtered with Phred quality score (minimum quality score > 10, mean quality score > 20), existence of ambiguous bases (no N) and read length (> 30 nt). Paired reads were identified using cmpfastq^3^.

### Genome-Guided Transcriptome Assembly

The clean reads were mapped to the carp reference genome with HISAT2 (v2.1.0) (Kim et al., 2015) under default parameters. The obtained SAM files were converted to BAM files and then sorted using SAMtools (v1.9) (Li, 2011). The sorted BAM files were assembled separately using StringTie (v1.3.4d) (Pertea et al., 2015) with default settings. Subsequently, the assemblies of each strain were merged into a single GTF file. The transcript sequences were extracted from the reference genome using gffread (v0.9.12)^4^.

### Differential Gene Expression Analysis

To identify the genes regulated by cold stressor, the samples (of the same tissue type and treatment) from different strains were regarded as biological replicates. A total of 4 groups, namely brain-ctrl, brain-CA, heart-ctrl, and heart-CA, each contained 6 samples, were included for the differential gene expression analyses. The clean reads were mapped to the reference transcriptome of carp using Salmon (v0.14.1) (Patro et al., 2017). The results of Salmon were processed using tximport to calculate gene/transcript abundance (transcript per minion, TPM) and counts of the raw reads mapped to each feature (Soneson et al., 2015). The raw reads count data sets were analyzed using DESeq2 (Love et al., 2014) to identify differentially expressed genes between Ctrl and CA of each tissue (Foldchange = 1.5, adjusted *p-*value = 0.05). Low abundance genes (number of summed reads < 10) were filtered before differential expression analysis. The DEG lists of brain and heart were compared using the online tool Venny (v2.1)^5^.

### Differential Splicing Analysis

The mapping results of STAR and the reference genome annotation file (V2.0.CommonC.gtf) downloaded from carpbase^6^ were provided to rMATS (v4.0.2) (Shen et al., 2014) to identify cold-regulated differential splicing events. Five types of alternative splicing events including skipped exon (SE), alternative 5′ splice site (A5SS), alternative 3′ splice site (A3SS), mutually exclusive exons (MXE) and retained intron were tested. IGVtools (v2.4.16) was used to visualize the DS analysis results and generate the sashimi plots.

### Quantitative Real Time PCR

Quantitative real-time PCR was performed as previously described (Long et al., 2015) to validate the results of RNA-seq using a CFX ConnectTM Real-Time PCR Detection System from BioRad. Each reaction included 5 μL of 2 × SYBER Green Real Time PCR Master mix (BioRad), 0.5 pmol of each primer and 2.5 μL of 10 × diluted cDNA templates in a total volume of 10 μL. The qPCR amplification program was 95°C 1 min, followed by 40 cycles of 95°C 10 s, 60°C 30 s (with plate read) and 72°C 10 s. The melt curve of PCR product was generated by heating from 65°C to 95°C with 0.5°C increments and 5 s dwell time, and a plate read at each temperature. The purity of reaction product was confirmed by the observation of a single melt peak. The amplification cycle displaying the first significant increase of the fluorescence signal was defined as threshold cycle and used for quantification (Cq). The primers were designed using Primer3Plus^7^. Standard curve of primer pairs was generated by analyzing the standard samples (serial dilutions of a mixture of all samples to be analyzed). Amplification efficiency of primers was calculated using slope of the standard curves. The sequence IDs, gene names, gene descriptions, amplification efficiency of primers, and the length of amplicons are listed in Supplementary Table 5.

To select internal reference genes for data normalization, the expression of 4 genes including *eef1g*, *naa50*, *med9*, and *nudcd2*, which demonstrated the most stable expression (based on RNA-seq data) across all the samples, was detected by qPCR and analyzed using geNorm (Vandesompele et al., 2002). The results indicated that *eef1g* and *naa50* was the most stable combination. Therefore, the geometric average of *eef1g* and *naa50* expression was calculated and used as the normalization factor for each sample. Three independent biological replicates for each treatment were included and all reactions were conducted in duplicates.

### Enrichment Analyses and Functional Classification of the CRGs

The carp genes were annotated to the orthologous genes of zebrafish and the corresponding zebrafish gene lists were used for the functional enrichment analyses. GO and KEGG enrichments for the DEGs were identified using the Cytoscape (v3.7.1) (Shannon et al., 2003) plugin ClueGO (Bindea et al., 2009). GO term fusion was applied to reduce the redundancy among the terms. List of all the expressed genes (TPM > 1 for both the replicates of at least one tissue and treatment) was used as the reference for the enrichment analyses. Function classification (protein class) was performed using PANTHER (14.1) (Mi et al., 2019) to gain insight into the function of the genes that underwent cold-induced differential splicing. Gene set enrichment analysis (GSEA) was performed using the GSEA (v4.1.0) (Subramanian et al., 2005) to identify the enriched functional gene sets from the total gene list.

### Total Cholesterol Concentration Measurement

Total cholesterol concentration was measured using the T-CHO kit (A111-1) from Nanjing Jiancheng Bioengineering Institute according to the manufacturer’s instruction. Briefly, the brain and liver samples of the Ctrl and CA fish were weighted and lysed on ice in 9-fold volume of ethanol. The lysate was centrifuged at 2500 rpm, 4°C for 10 min. The supernatant was transfer to a new tube and kept for subsequent measurement. Five μL of sample or standard was added to each well of 96-well plate, followed by 250 μL of reaction mix. Ethanol was used as blank for the measurement. After incubated at 37°C for 10 min, absorbance at 510 nm was measured by using a SpectraMax M5 microplate reader from Molecular Devices. Total cholesterol concentration (mmol/L) was calculated using the formula (sample*ABS* – blank*ABS*) ^∗^ 5.17/(standard*ABS* – blank*ABS*). Finally, total cholesterol content was normalized to the weight of tissues (μmol/g).

### Statistical Analysis

Statistical analyses were conducted using SPSS statistics (v22.0). The significance of difference between means of control and CA samples was analyzed with Independent-Samples *T*-tests.

## Data Availability Statement

The datasets presented in this study can be found in online repositories. The names of the repository/repositories and accession number(s) can be found in the article/Supplementary Material.

## Ethics Statement

The animal study was reviewed and approved by the Institutional Animal Care and Use Committee of Institute of Hydrobiology.

## Author Contributions

YL, XL, and ZC conceived the study and prepared the manuscript. ZQ, GG, and YL performed artificial reproduction and fish cultivation. YL, XL, FL, GG, and RL conducted sample collection, RNA extraction, and qPCR assay. YL measured total cholesterol content. YL and XL performed RNA-seq data analyses. QL and GS provided the experimental consumables. All authors read and approved the manuscript.

## Conflict of Interest

The authors declare that the research was conducted in the absence of any commercial or financial relationships that could be construed as a potential conflict of interest.

## Footnotes

1 http://www.ihb.ac.cn/fxcszx/

2 https://github.com/s-andrews/FastQC

3 http://compbio.brc.iop.kcl.ac.uk/software/cmpfastq.php

4 https://github.com/gpertea/gffread

5 https://bioinfogp.cnb.csic.es/tools/venny/

6 http://www.carpbase.org/

7 https://primer3plus.com/

## Supplementary Material

FIGURE S1 Sample correlation and results of the principle component analysis. **(A)** Heatmap of sample correlation matrix. The color bar represents correlation coefficient. **(B)** Result of principle component analysis (PCA).

FIGURE S2 Correlation between the results of RNA-seq and qPCR. The reference line indicates a significant correlation between the two variables (*p* = 1.46E-18).

FIGURE S3 Differentially expressed genes mapped to the spliceosome pathway. The up-regulated and down-regulated genes are shown in yellow and blue respectively.

FIGURE S4 Differentially expressed genes mapped to the steroid biosynthesis pathway. The up-regulated and down-regulated genes are shown in yellow and blue respectively.

TABLE S1 Reads filtering and mapping statics.

TABLE S2 List of differentially expressed genes.

TABLE S3 Genes demonstrating opposite responses to cold stress in brain and heart.

TABLE S4 Core cold responsive genes for common carp.

TABLE S5 Sequences of primers used for qPCR validation.
