# Supplementary figures and images for: Transcriptional Programs Underlying Cold Acclimation of Common Carp (Cyprinus carpio L.)
Source: Front Genet. 2020 Sep 23;11:556418. doi: 10.3389/fgene.2020.556418 (PMC7538616; doi:10.3389/fgene.2020.556418)

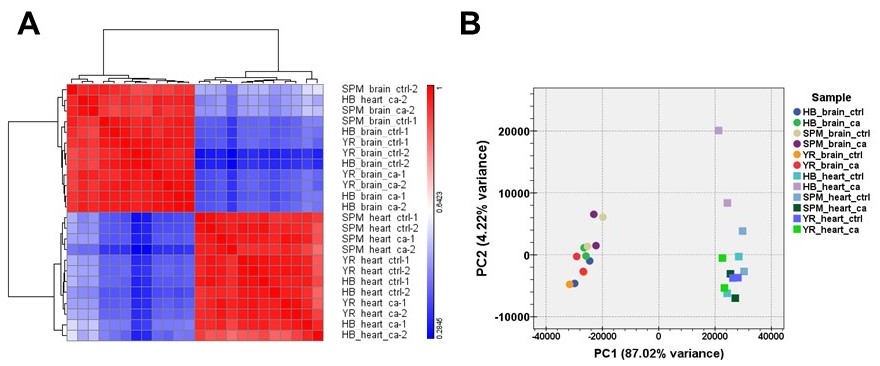

Supplement: FIGURE S1 — Sample correlation and results of the principle component analysis. (A) Heatmap of sample correlation matrix. The color bar represents correlation coefficient. (B) Result of principle component analysis (PCA). [file Image_1.JPEG]

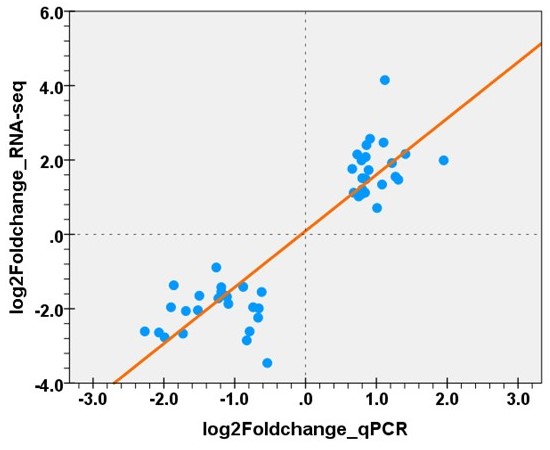

Supplement: FIGURE S2 — Correlation between the results of RNA-seq and qPCR. The reference line indicates a significant correlation between the two variables (p = 1.46E-18). [file Image_2.JPEG]

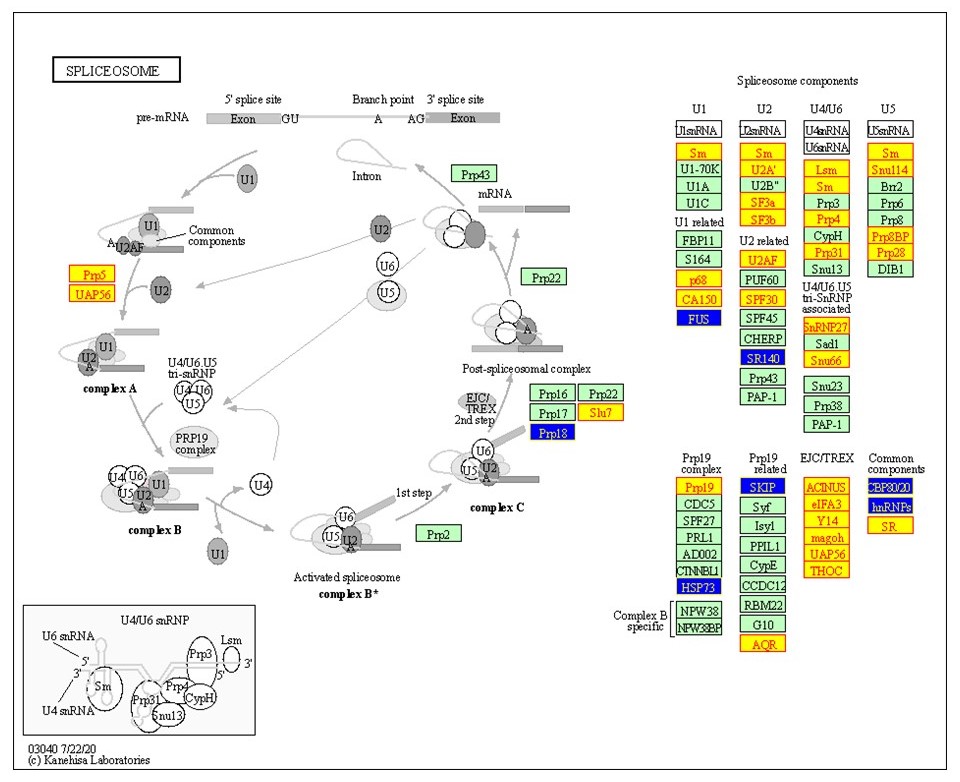

Supplement: FIGURE S3 — Differentially expressed genes mapped to the spliceosome pathway. The up-regulated and down-regulated genes are shown in yellow and blue respectively. [file Image_3.JPEG]

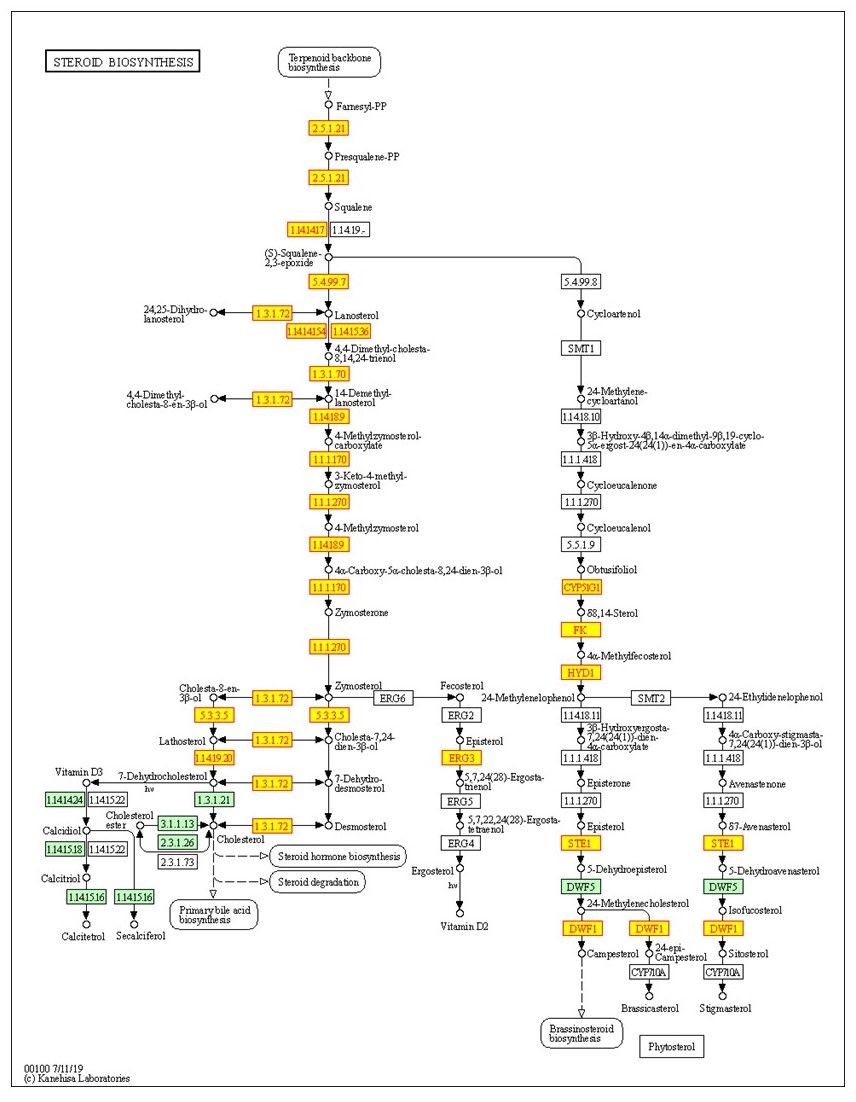

Supplement: FIGURE S4 — Differentially expressed genes mapped to the steroid biosynthesis pathway. The up-regulated and down-regulated genes are shown in yellow and blue respectively. [file Image_4.JPEG]
